# Supplementary figures and images for: The mitochondrial genome of the semi-slug Omalonyx unguis (Gastropoda: Succineidae) and the phylogenetic relationships within Stylommatophora
Source: PLoS One. 2021 Jun 25;16(6):e0253724. doi: 10.1371/journal.pone.0253724 (PMC8232460; doi:10.1371/journal.pone.0253724)

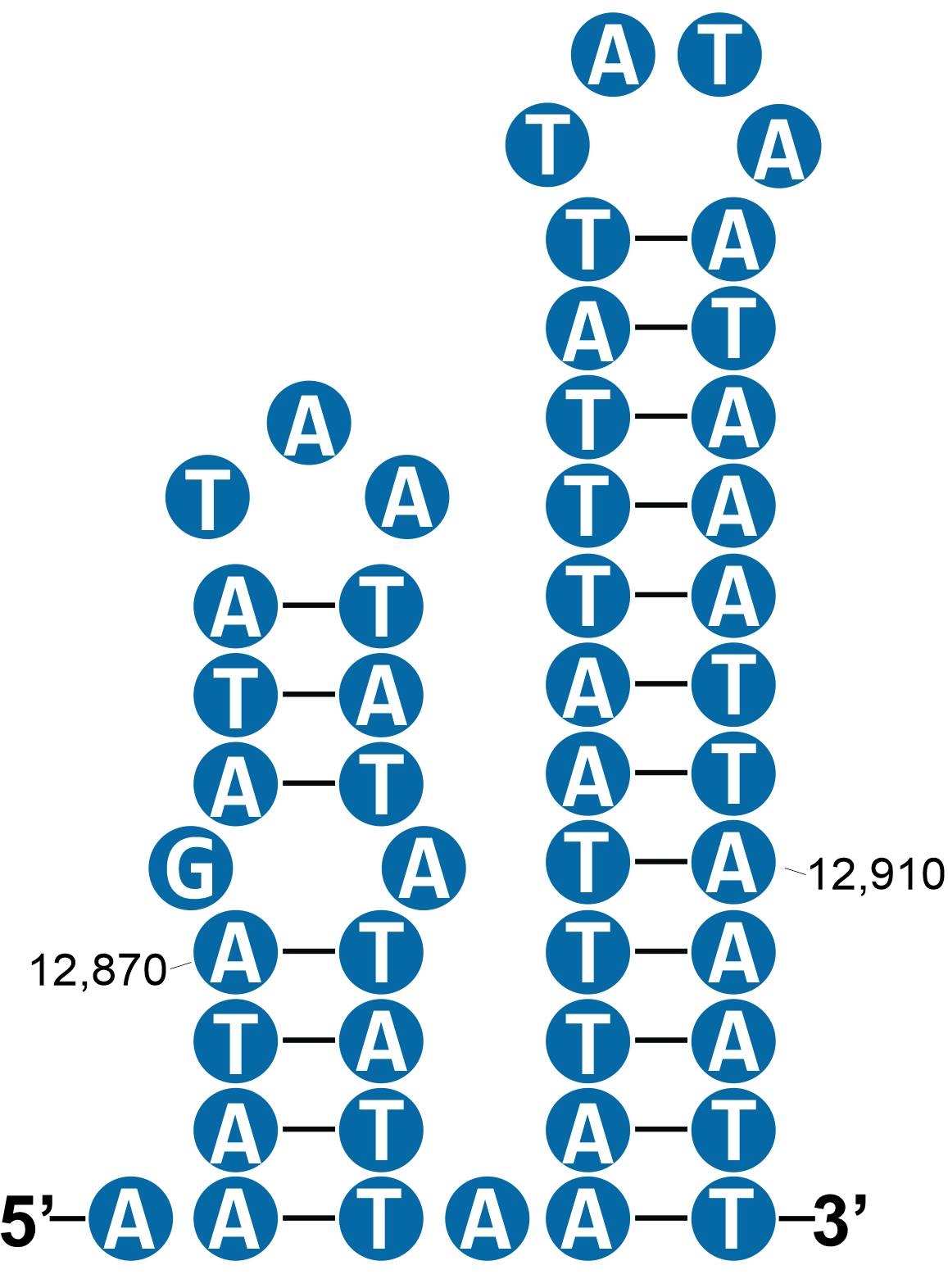

Supplement: S1 Fig — (TIF) [file pone.0253724.s001.tif]

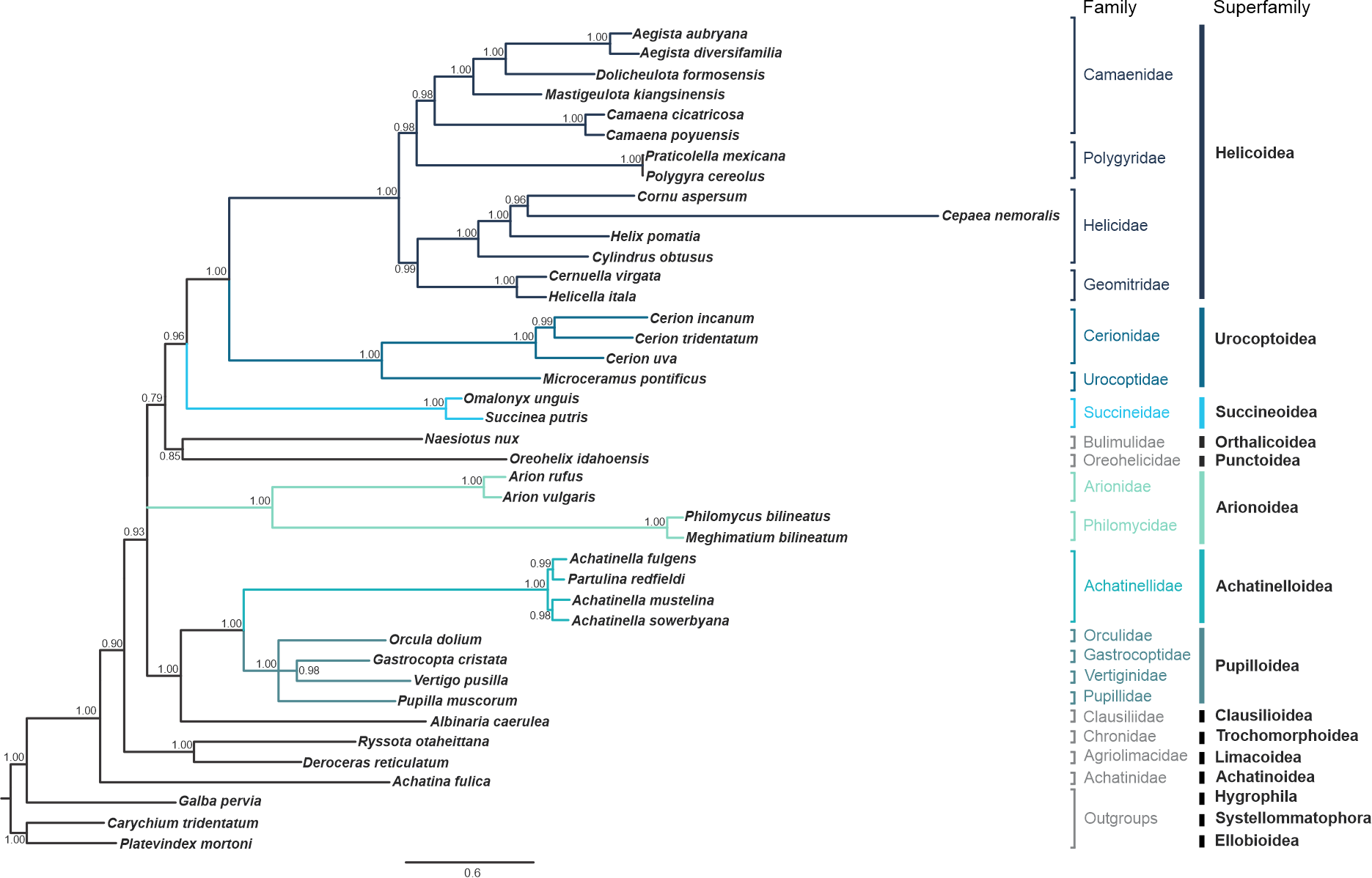

Supplement: S2 Fig — The trees were rooted with three outgroups (Carychium tridentatum, Platevindex mortoni and Galba pervia). The scale (0.6) shows evolutionary distances. Posterior probability support values are shown in the nodes. The GenBank accession numbers of the species are shown in Table 1; Omalonyx unguis was sequenced in this study. (TIF) [file pone.0253724.s002.tif]
