# Supplementary material for: The mitochondrial genome of the semi-slug Omalonyx unguis (Gastropoda: Succineidae) and the phylogenetic relationships within Stylommatophora
Source: PLoS One. 2021 Jun 25;16(6):e0253724. doi: 10.1371/journal.pone.0253724 (PMC8232460; doi:10.1371/journal.pone.0253724)
Supplement: S1 Table — Primer sequences are in the 5’ to 3’ direction. The primers naming system follows that of White et al. [39]. The genes encoded in the minus strand are underlined. (DOCX) [file pone.0253724.s003.docx]

**S1 Table.** Individual-specific primers of *Omalonyx unguis*. Primer sequences are in the 5’ to 3’ direction. The primers naming system follows that of White et al. [39]. The genes encoded in the minus strand are underlined.

| Gene | Primer | Sequence |
| --- | --- | --- |
| *cox1* | Ou-F1035 | GGGGGTTTAACTGGAATTGT |
| *rrnL* | Ou-R688 | ATTCCTGGGGTCTTCTCGTC |
| *rrnS* | Ou-F346 | ATGTGGTAAGTTCCCCTAAATG |
|  | Ou-R64 | CCCGTCGGTCTCAACTTTAAT |
| *tRNA^Trp^* | Ou-R25 | TGACCTTCAAAGCCATAAAAAGA |
| *atp6* | Ou-F169 | GCTAAGACAATATGTCCTGCTCTG |
| *nad4* | Ou-F895 | TTTCTCATGGTTTTATTTCACCTG |
| *cox3* | Ou-R168 | GCAACAGGATTTCACGGTCT |
