# Supplementary material for: The mitochondrial genome of the semi-slug Omalonyx unguis (Gastropoda: Succineidae) and the phylogenetic relationships within Stylommatophora
Source: PLoS One. 2021 Jun 25;16(6):e0253724. doi: 10.1371/journal.pone.0253724 (PMC8232460; doi:10.1371/journal.pone.0253724)
Supplement: S2 Table — RSCU: relative synonymous codon usage. (DOCX) [file pone.0253724.s004.docx]

**S2 Table.** Codon usage in *Omalonyx unguis*. RSCU: relative synonymous codon usage.

| Codon | Count | RSCU | Codon | Count | RSCU |
| --- | --- | --- | --- | --- | --- |
| UUU(F) | 393 | 1.91 | UAU(Y) | 150 | 1.74 |
| UUC(F) | 18 | 0.09 | UAC(Y) | 22 | 0.26 |
| UUA(L_2_) | 397 | 4.23 | CAU(H) | 50 | 1.54 |
| UUG(L_2_) | 39 | 0.42 | CAC(H) | 15 | 0.46 |
| CUU(L_1_) | 70 | 0.75 | CAA(Q) | 44 | 1.83 |
| CUC(L_1_) | 4 | 0.04 | CAG(Q) | 4 | 0.17 |
| CUA(L_1_) | 48 | 0.51 | AAU(N) | 153 | 1.84 |
| CUG(L_1_) | 5 | 0.05 | AAC(N) | 13 | 0.16 |
| AUU(I) | 360 | 1.91 | AAA(K) | 87 | 1.67 |
| AUC(I) | 17 | 0.09 | AAG(K) | 17 | 0.33 |
| AUA(M) | 217 | 1.81 | GAU(D) | 47 | 1.62 |
| AUG(M) | 23 | 0.19 | GAC(D) | 11 | 0.38 |
| GUU(V) | 113 | 2.31 | GAA(E) | 67 | 1.70 |
| GUC(V) | 5 | 0.10 | GAG(E) | 12 | 0.30 |
| GUA(V) | 68 | 1.39 | UGU(C) | 35 | 1.75 |
| GUG(V) | 10 | 0.20 | UGC(C) | 5 | 0.25 |
| UCU(S_2_) | 145 | 3 | UGA(W) | 70 | 1.67 |
| UCC(S_2_) | 16 | 0.33 | UGG(W) | 14 | 0.33 |
| UCA(S_2_) | 65 | 1.34 | CGU(R) | 15 | 1.15 |
| UCG(S_2_) | 5 | 0.10 | CGC(R) | 4 | 0.31 |
| CCU(P) | 75 | 2.52 | CGA(R) | 30 | 2.31 |
| CCC(P) | 12 | 0.40 | CGG(R) | 3 | 0.23 |
| CCA(P) | 31 | 1.04 | AGU(S_1_) | 61 | 1.26 |
| CCG(P) | 1 | 0.03 | AGC(S_1_) | 3 | 0.06 |
| ACU(T) | 87 | 2.49 | AGA(S_1_) | 79 | 1.63 |
| ACC(T) | 11 | 0.31 | AGG(S_1_) | 13 | 0.27 |
| ACA(T) | 39 | 1.11 | GGU(G) | 111 | 2.43 |
| ACG(T) | 3 | 0.09 | GGC(G) | 5 | 0.11 |
| GCU(A) | 70 | 2.28 | GGA(G) | 49 | 1.07 |
| GCC(A) | 12 | 0.39 | GGG(G) | 18 | 0.39 |
| GCA(A) | 39 | 1.27 |  |  |  |
| GCG(A) | 2 | 0.07 |  |  |  |
