# Supplementary material for: The mitochondrial genome of the semi-slug Omalonyx unguis (Gastropoda: Succineidae) and the phylogenetic relationships within Stylommatophora
Source: PLoS One. 2021 Jun 25;16(6):e0253724. doi: 10.1371/journal.pone.0253724 (PMC8232460; doi:10.1371/journal.pone.0253724)
Supplement: Attachment — Submitted filename: Response to Reviewers.pdf [file pone.0253724.s006.pdf]

MS PONE-D-21-05532R1

Title: **“The mitochondrial genome of the semi-slug *Omalonyx unguis* (Gastropoda: Succineidae) and the phylogenetic relationships within Stylommatophora”**

June 1, 2021

Dear Academic Editor
PLOS ONE
Ph.D. Brewer, Michael Scott

The MS was revised taking into account the comments suggested by you and the reviewers. The responses to the comments are detailed below.

Sincerely,

Lic. Leila B. Guzmán

Academic Editor

Thank you for submitting your manuscript to PLOS ONE. After careful consideration, we feel that it has merit but does not fully meet PLOS ONE’s publication criteria as it currently stands. Therefore, we invite you to submit a revised version of the manuscript that addresses the points raised during the review process.

Please pay particular attention to the minor issues raised by the reviewer.

We have introduced the suggested changes in the revised manuscript.

Reviewer No. 1

We thank Reviewer No. 1 for the positive comments on the revised manuscript and the suggestions provided.

Comments:

- **Lines 23-25: The sentence “Phylogenetic analyses based on the O. unguis mitogenome and 37 species of Stylommatophora were performed using amino acid sequences from the 13 protein-coding genes.” should be revised as “Phylogenetic analyses based on the mitogenomes of O. unguis and 37 other species of Stylommatophora were performed using amino acid sequences from the 13 protein-coding genes.” for the clarity.**

  We agree. We have incorporated the corrected sentence.

- **Line 32: “encodes 2 ribosomal RNAs” should be revised as “encodes two ribosomal RNAs”.**

  We agree. “2” was replaced by “two”.

- **In the legend of Figure 7 at lines 273-274: I would prefer to see the first letter of three-letter coded amino acids in capital.**

  We agree. We have added the first letter of three-letter coded amino acids in capital.
